# Mixed Psychological Changes Following Mastectomy: Unique Predictors and Heterogeneity of Post-traumatic Growth and Post-traumatic Depreciation

**DOI:** 10.3389/fpsyg.2017.01245

**Published:** 2017-07-20

**Authors:** Aleksandra Kroemeke, Kamilla Bargiel-Matusiewicz, Magdalena Kalamarz

**Affiliations:** ^1^Department of Psychology, SWPS University of Social Sciences and Humanities Warsaw, Poland; ^2^Department of Psychology, University of Warsaw Warsaw, Poland; ^3^Department of Psychology, Faculty in Katowice, SWPS University of Social Sciences and Humanities Katowice, Poland

**Keywords:** positive changes, negative changes, coping strategies, social support, breast cancer, mastectomy

## Abstract

**Objectives:** Post-traumatic growth (PTG) and its opposite—post-traumatic depreciation (PTD)—may be treated as important indicators of the patient quality of life. In the absence of studies on both, PTG and PTD in cancer patients, we investigated (1) coping strategies and support effectiveness as predictors of PTG and PTD in post-mastectomy women, (2) homogeneous classes with different intensity of PTG and PTD symptoms, and (3) correlates of class membership.

**Methods:** Coping strategies (Brief COPE), support effectiveness (SSE-Q), PTG (PTGI), and PTD (negatively reworded items of PTGI) were measured in 84 post-mastectomy women (mean age = 62.27, *SD* = 8.38). Multiple regression, two-step cluster, and multinomial logistic regression were applied.

**Results:** PTG and PTD had unique predictors: time since diagnosis and positive emotion-focused coping predicted PTG (*R*^2^ = 0.24), while negative emotion-focused and avoidance-focused coping and low support effectiveness were linked to PTD (*R*^2^ = 0.14). Four groups of PTG × PTD symptoms were identified: high PTG low PTD group (52.4%), low PTG low PTD group (17.9%), high PTG high PTD group (15.5%), and low PTG high PTD group (14.3%). Higher emotion- and avoidance-focused coping was characteristic for the high PTD low PTG group (*R*^2^ = 0.41).

**Conclusion:** Our findings shed light on the coexistence and unique predictors of PTG and PTD after mastectomy, indicating heterogeneity in PTG and PTD levels among post-mastectomy women.

## Introduction

Neoplastic disease and cancer-related treatment may significantly affect the well-being and quality of patient life. At the beginning, cancer research focused predominantly on the negative psychological changes, i.e., depression or distress (Epping-Jordan et al., 1999), but later studies revealed that cancer patients may also experience positive changes known as ‘post-traumatic growth (PTG)’ (Danhauer et al., 2013, 2015; McDonough et al., 2014). Although positive and negative perceived psychological changes in cancer continue to be investigated, only a few authors attempted to analyze them simultaneously (Schroevers et al., 2011). Furthermore, different approaches are often adopted, rendering it impossible to compare the findings. Thus, the existence of specific predictors for the perceived direction of change remains to be elucidated. In this study, we attempted to address this matter by testing whether perceived positive (post-traumatic growth, PTG) and negative (post-traumatic depreciation, PTD) changes in women after mastectomy have unique or shared predictors in terms of coping strategies and effectiveness of social support attempts called ‘social support effectiveness’ (SSE). Moreover, by applying a person-centered approach, which assumes that a population is heterogeneous in relation to the effect of the predictors on the outcome (Laursen and Hoff, 2006), we tested whether it was possible to identify homogeneous subgroups with particular intensity of both, PTG and PTD symptoms, and whether these subgroups differ in terms of coping strategies and SSE.

Negative changes in breast cancer (BC) women often go beyond the sphere of physical health, as the disease may also cause psychological problems and increase social demands. Various authors have demonstrated BC, and especially mastectomy, to be associated with negative feelings, including depressive symptoms, anxiety, distress (Epping-Jordan et al., 1999; den Heijer et al., 2012), post-traumatic stress symptoms (Hahn et al., 2015), lower quality of life (King et al., 2000; Janz et al., 2005), and changes in personal and social beliefs (Andrzejczak et al., 2013).

However, an accumulating body of evidence indicated that positive psychological effects may also occur during or after BC treatment (Sears et al., 2003; Bozo et al., 2009; Chan et al., 2011; Danhauer et al., 2013; Ruini et al., 2013; McDonough et al., 2014; Soo and Sherman, 2015). These positive changes, which are considered to be the result of coping with a traumatic event, are labeled as ‘PTG’ (Tedeschi and Calhoun, 2004). Data on the relationship between PTG and other well-being measures remain inconsistent (Sumalla et al., 2009; Ruini et al., 2013; Soo and Sherman, 2015). A recent meta-analysis of cancer patients revealed weak negative associations between PTG and depression and distress, weak positive associations with optimism, and no correlation with anxiety or physical quality of life (Shand et al., 2015), which calls into question the status of PTG: it remains unclear whether PTG and psychopathology constitute two ends of the same continuum or whether it is unrelated to adjustment. PTG has evoked much controversy regarding its nature and scope (Sumalla et al., 2009), chief among them the fact that most measuring tools include questions only about the positive (subjective) changes. As such, they fail to reflect the full scope of experiences and feelings of the respondents (Park and Lechner, 2014). Positive and negative experiences in any given set of circumstances, even within the same domain (e.g., social), are not mutually exclusive (Park and Lechner, 2014). Negative changes, which are considered to be the result of coping with a traumatic event, have been defined as PTD (Baker et al., 2008). Baker et al. (2008), designed a PTD measure based on the existing PTG assessment (PTGI; Tedeschi and Calhoun, 1996). Identical factors are involved in the measurement of both, perceived positive and negative changes as the effects of a stressful encounter, addressing the same domains and reducing the likelihood of positive or negative response bias (Baker et al., 2008). To the best of our knowledge, PTD in women with BC has not been investigated. Studies in other groups of patients demonstrated both constructs to co-exist (Purc-Stephenson et al., 2015), independently (Cann et al., 2010; Forgeard, 2013), and PTD to have good construct validity (Kunz et al., 2016).

Since PTG and PTD measure distinct and independent dimensions, it seems valid to check whether the conditioning mechanisms also differ. Perhaps separate factors are associated with positive and negative psychological changes. Successful identification of these factors might broaden our knowledge of PTG and PTG, resulting in more adequate preventive strategies. According to the abovementioned definition, these changes should be the result of coping with a traumatic event, which makes their relationship with the coping strategies particularly interesting. Various studies have indicated that in women with BC or after mastectomy, PTG is positively associated with problem–focused coping (active coping, planning, and seeking instrumental support) and positive emotion–focused coping (positive reframing, humor, seeking emotional support, turning to religion, and positive rumination), while being negatively related to negative emotion–focused coping (brooding) (Chan et al., 2011; Danhauer et al., 2013; Soo and Sherman, 2015). Similar findings have been reported by studies on different cancers (Schroevers et al., 2011; Tallman, 2013). Regardless, analyses including all coping strategies demonstrate that positive emotion–focused group (Schroevers et al., 2011) and problem–focused coping strategies (Tallman, 2013) were the best predictors. A recent meta-analysis of PTG correlates in 70 cross-sectional studies in cancer patients revealed its moderate relationship with positive reframing and religious coping (Shand et al., 2015). Similar dependence can be found in short-term (Scrignaro et al., 2011) and long-term longitudinal studies (Sears et al., 2003; Danhauer et al., 2013, 2015).

The relationship between PTD and coping remains to be fully elucidated as data are scarce. So far, the main focus has been on the relationship between PTD and rumination, indicating a positive link between PTD and intrusive rumination (Cann et al., 2010; Forgeard, 2013). In another study, PTD was positively related to self-distraction but unrelated to positive reframing, acceptance, and active coping (Schroevers et al., 2011). Taken together, the data suggest that negative perceived changes are related to negative emotion–focused and avoidance–focused coping.

Social support constitutes another important factor as far as positive and negative psychological changes in cancer patients are concerned (Shand et al., 2015). Social support is considered be to an important resource in the process of coping with adversity (Lazarus and Folkman, 1984) and, together with coping strategies, could be a significant predictor of PTG or PTD. Positive perceived changes in women with BC or post-mastectomy are linked with more perceived (Schroevers et al., 2010; Danhauer et al., 2013; Soo and Sherman, 2015) and received social support (Bozo et al., 2009; Scrignaro et al., 2011; McDonough et al., 2014). These effects were observed in a number of cross-sectional and longitudinal studies, and are supported by review (Helgeson and Cohen, 1996) and meta-analytic studies (Shand et al., 2015). Regardless, contradictory evidence has also been reported. According to a longitudinal study on predictors of PTG patterns by Danhauer et al. (2015), BC women with the highest as well as the lowest levels of PTG reported the highest perceived social support. Perceived or reported received support is not equivalent to received effective support (Rini and Dunkel-Schetter, 2010). Ineffective support may diminish the ability to cope with the diagnosis and treatment, and hinder adaptation to disease. Cancer patients who report the received support to be satisfactory adapt to cancer better (Manne and Badr, 2010). On the other hand, there is evidence that dissatisfaction with the received support did not predict cancer-related positive changes 8 years later (Schroevers et al., 2010). Nevertheless, our knowledge about SSE remains insufficient to determine whether it is related solely to adaptation or also to growth through adversity.

Our cross-sectional study attempted to address the abovementioned issues. We analyzed both, PTG and PTD within the same domains. As positive and negative perceived changes can coexist in cancer patients, their interrelationships were investigated. We expected that both constructs to be independent and that the population of women with BC or post-mastectomy to be heterogeneous in terms of perceived positive and negative psychological changes, i.e., different individuals may experience different proportion of both, positive and negative changes. This is in line with the person-centered approach. In contrast to variable-centered approach, where a population is assumed to be homogeneous in relation to the effect of predictor on the outcome, the person-oriented approach assumes that a population is heterogeneous in this respect (Laursen and Hoff, 2006). We also analyzed the mechanisms of differentiation for PTG and PTD. Specifically, the aims of the study were to investigate whether (1) coping strategies and SSE were specific or shared predictors of PTG and PTD in post-mastectomy women; (2) sample was heterogeneous in terms of PTG and PTD levels, and whether it was possible to identify homogeneous subgroups with different levels of both, PTG and PTD symptoms, and (3) these subgroups would differ in terms of the coping strategies and SSE. We hypothesized that PTG would be positively associated with problem-focused coping, positive-emotion focused coping and SSE, and negatively associated with negative-emotion-focused coping or avoidance coping, in contrast to PTD. The analysis of predictor character (specific or shared) was of an exploratory nature. We also hypothesized that several subgroups with different levels of PTG and PTD would be found, and that coping strategies and SSE would be linked do different PTG × PTD classes. Specifically, we expected that high problem-focused coping, positive-emotion focused coping and SSE and low negative-emotion-focused coping or avoidance coping to predict inclusion into high PTG subgroups. Opposite associations were expected for high PTD subgroups.

## Materials and Methods

### Participants and Procedure

The study included 84 women after mastectomy (age: 39–85 years, *M* = 62.27, *SD* = 8.38). The inclusion criteria were as follows: (1) history of BC, (2) history of radical or breast-conserving mastectomy, (3) no history of other major disabling medical or psychiatric conditions, and (4) age of ≥18 years. As far as education is concerned, 19% had primary, 56% secondary, and 25% higher education. As for marital status, 64.3% were married or cohabiting. Time since diagnosis (*M* = 10.20 ± 7.91 years) and surgery (*M* = 9.92 ± 7.99 years) ranged from 6 months to 34 years. Most women (85.7%) underwent single and radical mastectomy (single partial—9.5%; double partial—3.6%; double partial-radical—1.2%). Of all the women, 7.1% had breast reconstruction, and 66.7% perceived their treatment as completed.

This study was carried out in accordance with the recommendations of the SWPS University of Social Sciences and Humanities Ethics Committee with written informed consent from all participants. All participants gave written informed consent in accordance with the Declaration of Helsinki. The protocol was approved by the University Ethics Committee. A total of 120 women were invited to the study from the Polish Breast Cancer Associations, known as ‘the Amazons.’ The response rate was 70%.

### Measures

*Post-traumatic growth symptoms* were assessed using the 21-item Post-traumatic Growth Inventory (PTGI) (Tedeschi and Calhoun, 1996). Responses were provided on a 6-point scale, ranging from 0 (*I did not experience this change*) to 5 (*I experienced this change to a very great degree*). The overall PTG score was used (total score: 0–105). Higher scores reflected more PTG (α = 0.86).

*Post-traumatic depreciation symptoms* were assessed with 21 negatively worded items from PTGI (e.g., I *am less willing to express my emotions* as the negative alternative to the PTGI item *I am more willing to express my emotions*). Once the parallel scale to PTGI was designed, it was assessed in terms of wording by the expert and graduate students in psychology. Identical methodology was implemented by Baker et al. (2008). The participants used the same response scale and instruction as for PTGI. The overall PTD score was used (total score: 0–105). Higher scores indicated more PTD (α = 0.84).

*Coping strategies* were assessed with the abbreviated situational version of the COPE Inventory (Brief COPE) (Carver, 1997). The participants rated their behavior regarding BC and mastectomy on a 4-point scale ranging from 1 (*I haven’t been doing this at all*) to 4 (*I’ve been doing this a lot*). In its original form, the Brief COPE consists of 14 subscales (with only two items per scale). Due to low item reliability, and as per the suggestion of Carver et al. (1989), a second-order exploratory factor analysis was performed. Three higher-order factors were identified and further analyzed: problem–focused coping (active coping, planning, use of instrumental support; α = 0.74); positive emotion–focused coping (use of emotional support, positive reframing, acceptance, religion, humor; α = 0.61); and negative emotion– and avoidance–focused coping (venting, denial, substance use, behavioral disengagement, self-distraction, self-blame; α = 0.62). Higher scores reflected greater use of coping strategies.

*Effectiveness of social support attempts* was assessed with the Social Support Effectiveness Questionnaire (SSE-Q) (Rini and Dunkel-Schetter, 2010). The participants rated (a) whether the received amount matched the expected amount of support, (b) the extent to which they wished for different support, (c) whether support was provided in a skillful way, (d) the difficulty associated with getting support, (e) whether support was offered without asking, and (f) whether the received support resulted in negative effects (e.g., guilt). Points (a) to (e) were assessed on a 4-point scale from 0 (*very poor; not at all;* or *never;* depending on the content) to 4 (*excellent; extremely; always;* respectively), while negative effects were assessed on a two-point scale of 0 (*yes*) to 2 (*no*). Higher scores indicated greater SSE (α = 0.91).

### Statistical Analysis

Analyses were conducted using IBM SPSS (IBM Corp.; Armonk, NY, United States) ver. 23. Hierarchical multiple regression analysis was used to examine SSE and coping strategies as unique or shared predictors of PTG and PTD. An *a priori* power analysis using G-Power (Faul et al., 2007) was conducted to determine the minimum sample size required to detect small (*f* = 0.10) and medium (*f* = 0.26) effects with α = 0.05 and power = 0.80. The minimum acceptable sample size was determined to be *N* = 85 and *N* = 53, for small and medium effects, respectively. The demographic and health-related variables which correlated significantly with the outcomes were entered into the models as covariates. As the data contained missing values, the analysis of the missingness was performed first. Missing values of all raw data were <6% (5%—PTG, 4%—PTD, 5%—Brief COPE, 6%—SSE); Little’s MCAR test pointed to random missingness (χ^2^ = 3930.68, *df* = 4501, *p* = 0.999). Missing data were imputed, according to recommendations, using the expectation-maximization method, which is an iterative procedure which produces maximum likelihood estimates (Graham, 2009).

To identify homogeneous subgroups with different levels of both PTG and PTD symptoms, a two-step cluster analysis was conducted. The minimum recommended sample size for this analysis is 5 × 2^k^, where *k* is the number of the variables in the analysis (Dolnicar, 2002). According to recommendations (Sarstedt and Mooi, 2014), the best solution was chosen based on Bayesian and Akaike information criterion (BIC and AIC, respectively) indicators, the ratio of distance measure, and the Silhouette measure of cluster cohesion and separation, as well as interpretation opportunities and theoretical integrity of the selected solution. The results of automatic determination of the number of classes were also taken into consideration; however, this procedure is based solely on the ratio of distance measures (Arbuckle, 1995). The model with lower criterion indicators and the greatest ratio of distance measure and cohesion (>0.50) was preferable. Subsequently, correlates of subgroup membership were tested. Multinomial logistic regression analysis (MLR) was conducted to determine the relationship between demographic and health-related variables, coping strategies, SSE, and inclusion in the PTG × PTD subgroups. An *a priori* power analysis using G-Power (Faul et al., 2007) indicated that the minimum acceptable samples size for this analysis is from *N* = 53 (for OR = 3) to *N* = 721 (for OR = 1.3) with probability = 0.03, α = 0.05 and power = 0.80. In MLR, the goodness of fit was assessed using Pearson’s χ^2^; the parameters were estimated using the likelihood ratio (χ^2^ statistics) for the whole model and the Wald *Z*-statistic value and the odds ratio (OR) for each independent variable.

## Results

Descriptive statistics and correlations between the variables are presented in **Table 1**. The level of PTG was significantly higher as compared to PTD (*t*_83_ = 14.69, *p* < 0.001). PTG and PTD were not significantly correlated.

**Table 1:** Descriptive statistics and correlations (*N* = 84).

Variable	*M*	*SD*	2	3	4	5	6
(1) PTG	74.83	16.58	0.08	0.32^∗∗^	0.41^∗∗^	0.02	0.06
(2) PTD	30.17	21.96		0.05	0.08	0.34^∗∗^	-0.33^∗∗^
(3) PC	5.63	1.19			0.57^∗∗^	0.51^∗∗^	-0.03
(4) PEC	5.22	0.91				0.36^∗∗^	-0.13
(5) NEAC	4.88	0.88					-0.32^∗∗^
(6) SSE	53.19	13.60					


*PTG, post-traumatic growth; PTD, post-traumatic depreciation; PC, problem-focused coping; PEC, positive emotion-focused coping; NEAC, negative emotion- and avoidance-focused coping; SSE, social support effectiveness. ^∗∗^*p* < 0.01.*

### Predictors of PTG and PTD

Multiple regression analysis (**Table 2**) showed that higher PTG was predicted by higher positive emotion–focused coping and longer time since diagnosis. Higher PTD was related to higher emotion– and avoidance–focused coping and lower SSE.

**Table 2:** Multiple regression analysis predicting PTG and PTD.

	PTG						PTD					
		
	*B*	*SE*	β	*Adj.R*^2^	Δ*R*^2^	*F*	*B*	*SE*	β	*Adj.R*^2^	Δ*R*^2^	*F*
**Step 1^a^**												
Age	-0.38	0.25	-0.19									
Time since diagnosis	0.42	0.27	0.20									
Education				0.02		*1.65*	-7.70	3.40	-0.24*	0.05		5.14^∗^
**Step 2**												
Age	-0.39	0.23	-0.19									
Time since diagnosis	0.58	0.26	0.27*									
Education				0.22	0.24^∗∗∗^	4.82^∗∗∗^	-3.98	3.43	0.13	0.15	0.14^∗^	3.84^∗∗^
PC	2.61	1.86	0.19				-1.95	2.55	-0.11			
PEC	7.36	2.25	0.40**				-0.44	3.05	-0.02			
NEAC	-2.52	2.45	-0.13				7.45	3.21	0.30*			
SSE	0.13	0.14	0.10				-0.35	0.18	-0.22*			


*PTG, post-traumatic growth; PTD, post-traumatic depreciation; PC, problem-focused coping; PEC, positive emotion-focused coping; NEAC, negative emotion- and avoidance-focused coping; SSE, social support effectiveness. Age, Time since diagnosis, Education: the higher score, the higher age/time since diagnosis/education. ^a^Only covariates significantly related to the outcomes in prior analyses were entered into the models.^∗^*p* < 0.05, ^∗∗^*p* < 0.01, ^∗∗∗^*p* < 0.001.*

### Different Classes of PTG and PTD and Their Correlates

**Table 3** shows the fit indicators for PTG and PTD class solutions in a two-step cluster analysis. The BIC value and ratio of distance measures supported the 3-class solution; the automatic determination of the number of classes also indicated this model. The AIC value supported the 5-class model. The Silhouette measure of cluster cohesion and separation revealed good fit for models with 3, 4, and 5 clusters (all >0.50). However, AIC overestimates and BIC slightly underestimates the correct number of clusters (Sarstedt and Mooi, 2014). The 4-class solution was chosen, which is consistent with the recommendations of Sarstedt and Mooi (2014), given that it was characterized by satisfactory AIC and BIC values (*k*—1 class compared with the lower values), and the best goodness of fit of the model and the second-largest ratio of distance measures.

**Table 3:** Two-step cluster analysis of PTG and PTD—fit indexes.

Number of classes	Average Silhouette value	AIC	BIC	Ratio of distance measure	Class size (*n*)
1		123.45	133.17		84
2	0.40	99.19	118.64	1.20	25/59
3	0.50	80.41	109.58	2.02	22/47/15
4	0.60	75.13	114.07	1.57	15/44/13/12
5	0.50	74.74	123.36	1.38	15/22/23/12/12


In this solution, most of the sample (*n* = 44, 52.4%) belonged to the group with high PTG and low PTD (*M* = 83.48 ± 7.46 for PTG; *M* = 20.95 ± 11.82 for PTD). The second group (*n* = 15, 17.9%), included women low PTG and very low PTD (*M* = 55.99 ± 13.13 for PTG; *M* = 11.61 ± 7.62 for PTD). In the third group (*n* = 13, 15.5%), participants showed relatively high PTG and PTD (*M* = 84.59 ± 9.37 for PTG; *M* = 67.05 ± 13.52 for PTD). The last class (*n* = 12, 14.3%) comprised participants with relatively low PTG and high PTD scores (*M* = 56.13 ± 16.29 for PTG; *M* = 49.79 ± 6.40 for PTD).

Demographic and health-related variables did not show any significant relation to class membership. The results of MLR after controlling for age, education, and time since diagnosis (**Table 4**) revealed that emotion– and avoidance–focused coping differentiated the class membership, with the high PTD low PTG group using more of these strategies as compared to the low PTG low PTD group and the high PTG low PTD group.

**Table 4:** Multinomial logistic regression predicting different PTG × PTD clusters.

	*B*	*SE*	Wald χ^2^	OR	95% Cl OR	
					
					lower	Upper
**Reference category: *High PTD low PTG group***						
*Low PTG low PTD group*						
Intercept	-1.24	5.55	0.05			
Age	0.01	0.06	0.02	1.01	0.89	1.14
Time since diagnosis	-0.08	0.08	0.86	0.93	0.79	1.09
Education^a^	-0.52	1.20	0.18	0.60	0.06	6.28
PC	1.05	0.68	2.40	2.87	0.76	10.23
PEC	0.93	0.76	1.51	2.54	0.57	11.23
NEAC	-2.40	0.85	7.95^∗∗^	0.90	0.02	0.48
SSE	0.06	0.04	2.12	1.07	0.98	1.16
*High PTG low PTD group*						
Intercept	1.11	4.54	0.06			
Age	-0.03	0.05	0.37	0.97	0.87	1.07
Time since diagnosis	-0.02	0.07	0.08	0.98	0.85	1.13
Education^a^	-0.40	1.08	0.13	0.68	0.08	5.59
PC	1.23	0.63	3.81	3.43	1.00	11.82
PEC	0.66	0.67	0.98	1.94	0.52	7.19
NEAC	-1.92	0.76	6.33^∗^	0.15	0.03	0.65
SSE	0.04	0.04	1.54	1.05	0.97	1.12
*High PTG high PTD group*						
Intercept	-5.30	5.97	0.79			
Age	0.01	0.07	0.04	1.01	0.88	1.16
Time since diagnosis	-0.09	0.10	0.94	0.91	0.754	1.10
Education^a^	-1.13	1.55	0.54	0.32	0.02	6.68
PC	1.17	0.73	2.58	3.11	0.77	13.41
PEC	1.25	0.77	2.60	3.48	0.76	15.91
NEAC	-1.48	0.89	2.87	0.23	0.04	1.26
SSE	0.00	0.04	0.00	1.00	0.92	1.09
**Reference category: *High PTG high PTD group***						
*Low PTG low PTD group*						
Intercept	4.06	5.90	0.47			
Age	-0.00	0.06	0.00	1.00	0.88	1.13
Time since diagnosis	0.02	0.09	0.04	1.02	0.86	1.20
Education^a^	0.61	1.33	0.21	1.85	0.14	24.92
PC	-0.11	0.52	0.05	0.98	0.32	2.50
PEC	-0.32	0.06	0.27	0.73	0.22	2.38
NEAC	-0.02	0.70	1.77	0.39	0.10	1.55
SSE	0.06	0.04	2.34	1.06	0.98	1.15
*High PTG low PTD group*						
Intercept	6.42	4.95	1.68			
Age	-0.05	0.05	0.75	0.95	0.86	1.06
Time since diagnosis	0.07	0.07	0.98	1.08	0.93	1.24
Education^a^	0.74	1.21	0.37	2.10	0.20	22.49
PC	0.06	0.47	0.02	1.07	0.43	2.66
PEC	-0.59	0.53	1.24	0.56	0.20	1.56
NEAC	-0.44	0.59	0.56	0.64	0.20	2.05
SSE	0.04	0.93	1.70	1.04	0.98	1.11
**Reference category: *High PTG low PTD group***						
*Low PTG low PTD group*						
Intercept	-2.35	4.26	0.30			
Age	0.04	0.04	0.89	1.04	0.95	1.14
Time since diagnosis	-0.06	0.05	1.11	0.95	0.85	1.05
Education^a^	-0.13	0.73	0.03	0.88	0.21	3.72
PC	-0.18	0.35	0.26	0.84	0.42	1.65
PEC	0.27	0.46	0.35	1.31	0.53	3.22
NEAC	-0.48	0.48	1.01	0.62	0.24	1.58
SSE	0.02	0.03	0.41	1.02	0.96	1.08


*Likelihood ratio test: for the model, χ^*2*^(21) = 38.09, *p* = 0.013; problem-focused coping (PC), χ^*2*^(3) = 5.09, *p* = 0.166; positive emotion-focused coping (PEC), χ^*2*^(3) = 3.32, *p* = 0.344; negative emotion- and avoidance-focused coping (NEAC), χ^*2*^(3) = 11.03, *p* = 0.012; social support effectiveness (SSE), χ^*2*^(3) = 3.80, *p* = 0.284; age, χ^*2*^(3) = 1.55, *p* = 0.670; time since diagnosis, χ^*2*^(3) = 2.33, *p* = 0.508; education, χ^*2*^(3) = 0.62, *p* = 0.892. Goodness-of-Fit: Pearson χ^*2*^(222) = 217.25, *p* = 0.577; Nagelkerke Pseudo *R*^2^ = 0.41. ^a^Reference category: higher education. ^∗^*p* < 0.05, ^∗∗^*p* < 0.01.*

## Discussion

The aim of the study was to investigate the similarities and differences between PTG and PTD predictors in women after mastectomy. The heterogeneity in the perceived changes, i.e., identification of homogeneous classes with different PTG and PTD scores and class membership correlates, was also addressed. The following significant unique predictors were revealed: time since diagnosis and positive emotion–focused coping were predictors for PTG, while emotion– and avoidance–focused coping and low SSE were predictors for PTD. The study participants were subdivided into four classes, based on the interaction of high *vs*. low PTG and PTD. However, only emotion–focused coping differentiated class membership.

Post-traumatic growth and PTD were found to be uncorrelated, which is consistent with the findings of earlier studies (Cann et al., 2010; Forgeard, 2013). The results suggest these variables to be independent dimensions, with separate trajectories of development in the cognitive and behavioral sphere. They also are consistent with data on the independence of positive and negative affect (Larsen and McGraw, 2011), or positive and negative processes, thus supporting the theory of multipolar rather than bipolar adjustment properties. In consequence, coping with traumatic events might result in both, growth and depreciation, or either, or neither.

Another finding of our study is that fact that not all women experienced psychological changes following mastectomy in the same manner. Four different classes of change were identified in the post-mastectomy patients. A little over half of the women experienced only growth, with the remaining subjects reporting no change, mixed perceived changes, or only negative perceived changes. Interestingly, an analysis of the entire sample (based on mean scores according to a variable-centered perspective where the assumption is homogeneous of population (Laursen and Hoff, 2006) revealed that the affected women had higher PTG than PTD, which may indicate excellent adaptation to mastectomy. However, this does not account for approximately 14% of women with only PTD, 15.5% with both, PTG and PTD, and 18% with neither PTG nor PTD. In fact, psychological changes following mastectomy varied with each individual, indicating that the group lacked homogeneity in terms of variables. Taking that into account, it is possible to avoid the mistake of psychological change-related generalizations about the entire group. It also provides proof that PTD is not a simple opposite of PTG and that both these variables constitute largely independent dimensions.

The first group had high PTG and low PTD scores, signifying the highest potential for personal development in that group. Also, these women were probably the most motivated to introduce changes in their personal life, including compliance with the doctor’s orders and consistent care about their health and well-being. The second group had low PTG and low PTD scores. That group experienced the least significant consequences at the cognitive-emotional level. No perceived change might indicate ‘hedonic adaptation’ or ‘satisfaction treadmill’, i.e., returning to baseline, pre-trauma well-being (Lyubomirsky, 2011). Women from the third group had relatively high PTG and high PTD, and were most probably in the process of attaining mental balance. As such, they might alternately experience symptoms of PTG and PTD. Their scores indicate significant dynamics of the cognitive-emotional processes. An analysis of the mean results in the subgroups showed that the group with mixed perceived changes had the highest PTG and PTD scores. Coexisting PTG and PTD provide evidence for the high complexity of the coping processes, and the need for a comprehensive analysis of the potential changes, for example using the person-centered approach (Laursen and Hoff, 2006). The results might offer supporting arguments for the debate on the real vs. illusory facet of PTG (Maercker and Zoellner, 2004; Zoellner et al., 2008; Frazier et al., 2009; Pat-Horenczyk et al., 2015; Lahav et al., 2016). Women from the fourth group had low PTG and high PTD, and experienced the highest level of discomfort. The perception of high PTD, together with relatively low PTG symptoms might be considered a proof of the positive–negative asymmetry, i.e., negativity outweighing positivity (Baumeister et al., 2001).

Another finding to support the independence of PTG and PTD was that their correlates—in terms of coping strategies and SSE—were unique. Higher positive emotion–focused coping was indicative of PTG, while negative emotion–focused and avoidance–focused coping, as well as low SSE, predicted PTD, after controlling for demographic and medical-related factors, which is consistent with earlier studies (Cann et al., 2010; Schroevers et al., 2011; Forgeard, 2013; Tallman, 2013). However, these results do not support data on the relationship between PTG and problem–focused coping. Thus, it seems safe to conclude that both ‘results of coping’ had their own, specific determinants. As expected, PTG was positively related to behaviors such as positive reframing, acceptance, and religion, which was confirmed in a previous meta-analysis (Shand et al., 2015). Such behaviors, often referred to as ‘adaptive,’ allow for a different perception of traumatic events. The affected patients perceive them as meaningful. PTD, in turn, was positively correlated with maladaptive strategies, i.e., evoking or supporting negative emotions or diverting one’s attention from a problematic situation, for example by denial. Interestingly, only maladaptive strategies differentiated homogeneous group membership with different PTG and PTD scores. Such behavior pattern was characteristic for women with low PTG and high PTD, thus allowing to predict depreciation rather than growth. Our findings are consistent with earlier studies which confirmed the existence of the negativity bias (Baumeister et al., 2001), implying that negativity is more powerful and thus manifests itself more strongly.

Social support effectiveness effects may be treated in a similar manner. Low effectiveness of social support attempts positively correlated with the negative outcomes, i.e., PTD, which is consistent with earlier findings (Manne and Badr, 2010; Rini and Dunkel-Schetter, 2010). It suggests that SSE is more closely related to the return to baseline well-being (adaptation) or with worsening function (depreciation) than growth. However, a comparison of homogeneous groups did not support that hypothesis. The role of SSE in the coping process requires further analysis.

As for background variables, longer time since diagnosis was associated with PTG, which is consistent with other reports (Sears et al., 2003; Tedeschi and Calhoun, 2004; Danhauer et al., 2013). No relationship was found between demographic and medical-related variables and homogeneous subgroup membership. Our results might also be related to other variables which are relevant to post-traumatic changes, such as event centrality (Johnson and Boals, 2014) or self-efficacy (Hobfoll et al., 2007), but which were not accounted for in this study.

Our study is not without limitations. Due to the cross-sectional design of the study, we were not able to draw conclusions about the cause-and-effect relationships between the investigated variables. Furthermore, we lacked data on the pre-cancer levels of well-being of the study participants. There was also a relatively large variability in terms of time since diagnosis and time since mastectomy. Furthermore, the evaluation of the changing direction in growth and depreciation was subjective and, thus, distorted. The shortcomings of PTGI are well-known (Frazier et al., 2009), and the same applies to the measurement of PTD. Moreover, the sample size, especially in case of the MLR analyses, could have been insufficient, resulting in underpowered results and failure to identify significant effects. These limitations, along with the lack of analyses of specific domains of PTG and PTD, indicate the necessity of further studies.

Despite these limitations, we are of the opinion that our findings have cast new light on the understanding and practical implications of positive and negative perceived changes following mastectomy. The results may lead to the development of more tools to provide adequate psychological support for women after mastectomy, with special focus on the reduction of maladaptive coping strategies. Apart from developing strategies to prevent PTD or reduce its intensity, our study may raise awareness about the diversity of psychological reactions in patients, thus preventing stereotypical and routine approach to their problems. Particularly women from the mixed (high PTG/PTD) and the high PTG groups should receive psychological care and counseling. The former are most probably in the process of attaining mental balance and, thus, ought to be offered access to a psychologist who would facilitate constructive changes in their functioning. The latter, on the other hand, experience the highest level of mental discomfort and, as such, ought to receive psychological care and psychiatric consultation.

## Author Contributions

Substantial contributions to the conception or design of the work (AK); or the acquisition, analysis (AK), or interpretation of data for the work (AK, KB-M, and MK). Drafting the work or revising it critically for important intellectual content (AK, KB-M, and MK). Final approval of the version to be published (AK, KB-M, and MK). Agreement to be accountable for all aspects of the work in ensuring that questions related to the accuracy or integrity of any part of the work are appropriately investigated and resolved (AK, KB-M, and MK).

## Conflict of Interest Statement

The authors declare that the research was conducted in the absence of any commercial or financial relationships that could be construed as a potential conflict of interest.
